# Micro-Image Strain Sensing Method for Displacement and Strain Measurement in One United Sensor

**DOI:** 10.3390/s23010397

**Published:** 2022-12-30

**Authors:** Lixiao Zhang, Xixian Chen, Weijie Li, Botao Xie, Guangyi Zhou, Xuefeng Zhao

**Affiliations:** 1 School of Civil Engineering, Dalian University of Technology, Dalian 116024, China; 2 State Key Laboratory of Coastal and Offshore Engineering, Dalian University of Technology, Dalian 116024, China; 3 College of Civil Engineering and Architecture, Wenzhou University, Wenzhou 325035, China; 4 Northeast Branch of China Construction Eighth Engineering Bureau Division Corp, LTD., Dalian 116021, China

**Keywords:** one united sensor, improved MISS method, strain and displacement, structural health monitoring, image processing

## Abstract

Micro-image strain sensing (MISS) is an innovative technology to measure strain within a measurement range of ±8300 microstrains. It has been proved to be effective and satisfy all requirements in the field of structural health monitoring. However, there is still room for improvement and extend the range of measurement. In this paper, an improved method is proposed to increase the measurement range of strain and displacement. Moreover, various tests were conducted to verify the efficiency of the improved method. The results showed that the modified method is efficient and accurate and can be readily used to extend the measurement range of both strain and displacement. This research will likely help stimulate the development of multifunctional sensors to obtain abundant useful information about structures in the field of structural health monitoring. It will allow measuring strain and displacement, which require different levels of accuracy, using one unified sensor.

## 1. Introduction

Structural health monitoring aims to obtain useful information for the safety and maintenance of infrastructures. Strain or displacement are key elements to evaluate the health status of structures and predict their remaining service life in the field of structural health monitoring. Anomalies can be found in time, allowing taking the necessary measures, by monitoring strain and displacement, and the deformation mechanisms can be clearly understood to establish better engineering theories and methods [1].

Strain is a relative change in the deformation of an object in the direction of an applied force and is therefore a vector quantity. Several techniques have been developed for in-plane strain measurements based on different principles; they have both advantages and disadvantages. Strain gauges have even been widely used because of their simplicity, high accuracy, and low cost [2]. However, they have limitations in terms of sensing, due to their relatively small size and specialized requirements. Although interferometric metrology can provide full-field deformation measurements with high accuracy, it requires a coherent light source and a vibrational isolation platform [3]. Moreover, fiber optic strain sensors, especially, fiber Bragg grating (FBG) sensors, have been successively applied to bridges, reinforced concrete and composite structures [4,5,6]. The digital image correlation (DIC) method has been widely used as a powerful and practical tool for surface deformation measurement and directly provides full-filed displacements and strains by comparing the captured images before and after deformation [7,8,9,10]. Nevertheless, the DIC technology depends heavily on the quality of the images, and its accuracy is lower than that of interferometric metrology.

Displacement is defined as the change of the deformation of an object from the initial position to the final position and is a scalar quantity. Various techniques for measuring surface displacement have been developed to estimate the structural behavior, such as displacement sensors, laser technology and vision-based technology [11,12,13,14,15]. There are different types of displacement sensors, which are applicable on a large scale for defect detection and deformation measurement in civil engineering [16]. For instance, capacitive displacement sensors are designed to measure relative long-range displacements with high accuracy [17]. However, their measurement range is limited to about 20 mm, and they are susceptible to disturbance from the environment. The displacement measurement based on laser technology is quite mature and has the advantage of high precision [18,19,20]. Nevertheless, in this approach, the light source can be easily blocked, which makes it difficult to widely employ this technique in real engineering. In addition, the sampling moiré method has been widely used to measure displacement and strain [21]. Vision-based technology is becoming more refined and more acceptable in the field of strain and displacement measurement [22,23]. This is a non-contact measurement method, which has the advantages of being non-invasive and non-destructive to the structure.

The measurement methods for displacement and strain are various and well established. However, there has been limited research on measuring strain and displacement with a unified sensor technology, which is mainly caused by the different measurement ranges and accuracies required by the two types of measurement. Micro-image strain sensing (MISS) based on computer vision has been developed to measure strain. This is a cheap, convenient and universally applicable strain measuring method. Our previous research concluded that the maximum measurement range of this technique is ±8300 microstrains [24,25,26]. A study [24] proposed the SURF method as a strain measurement method to trace the displacement of feature points in micro-images and obtain the strain in objects. Reference [25] verified the accuracy of the proposed method with different pixel values and different distances. Another study [27] tested the static and dynamic performance of the proposed method. In addition, an improved method for long-term monitoring was developed by using a digital microscope camera instead of a smartphone in reference [28], and a comparative study of the MISS sensor and FBG sensor was provided in reference [6]. Moreover, the application of a micro-image strain sensing (MISS) sensor based on a smartphone and a microscope for measuring structural members was investigated [29].

In the present study [28], an approach to increase the measurement range is presented with the aim of obtaining information on strain and displacement using one unified sensor. Such a combined approach has not yet been reported in the literature, and this research could provide a developmental continuum between studies on the measurement of strain and displacement. This approach can measure the displacement at both macroscopic and microscopic levels with high accuracy, which makes it more widely applicable for structural health monitoring.

## 2. The MISS Method and Its Shortcoming

### 2.1. The Principle of the MISS Method

**Figure 1:**
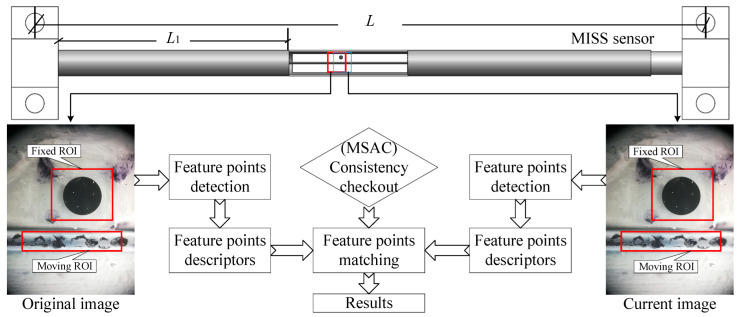
The process diagram of the MISS method based on the SURF and MSAC algorithms. The fixed region of interest (ROI) represents a fixed area is fixed, and the moving ROI can move horizontally. *L* represents the length of the MISS sensor, and *L*_1_ is the length of the left tube.

The MISS method for strain measurement is based on the speeded up robust features (SURF) algorithm. SURF is a subpixel image registration algorithm and is used to detect the feature points [30]. This algorithm is well immune to variations in brightness and image noise. Moreover, M-estimator sample consensus (MSAC) is adopted to remove outliers for improving the accuracy [31]. The flow chart of feature point matching is shown in Figure 1, and the following describes the main steps of the MISS method.

(1) Read all the images in the folder in sequence and select the first one as the reference image A.
(2) Determine the fixed region of interest (ROI) of image A where there is a known circle (its diameter was 1 mm in this study). Calculate the proportion K between pixel distance and actual distance according to the circle.
(3) Determine the moving ROI of image A and assign image B to all images in the folder.
(4) The SURF algorithm is used to match feature points in A and B. Moreover, the MSAC algorithm is adopted to correct the wrong matching points.
(5) Obtain the average deformation of each image B in pixel coordinates. Utilizing the deformation, strain can be calculated based on the proportion *K* and the length *L*.
(6) Output and save the strain data to a file.

### 2.2. Shortcomings of the MISS Method

The MISS method, combining a smartphone and a portable microscope, has been verified to have high precision and high stability and thus satisfies the requirement of structural health monitoring in civil engineering [27]. In a previous work, the diameter of the captured micro-images was about 2.5 mm, which means that movement could not be identified if it exceeded ±2.5 mm. In the above steps, the first image is selected as the reference image for feature points matching. Nevertheless, once a large deformation occurs, the feature points in the reference image cannot be observed. In such circumstances, the SURF program will fail and terminate.

## 3. Improved MISS Method

**Figure 2:**
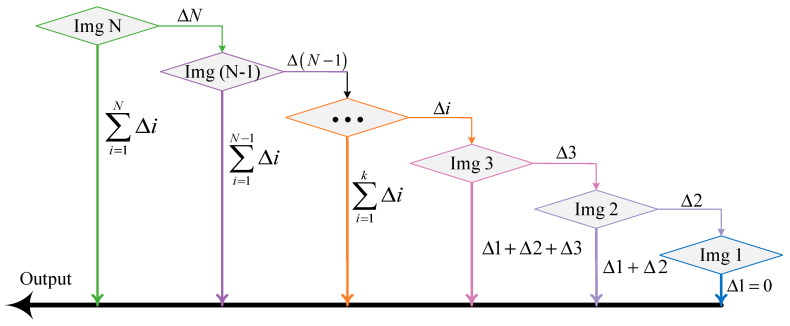
Process of the MISS1 method; ∆i represents the movement from an image to the next image. For Img *i*, the movement is the sum of the lengths from ∆1 to ∆i.

This section describes the improved MISS method, which mainly aims at increasing the measurement range without decreasing the accuracy and develops a technique based on the measurement of macroscopic strain and microscopic displacement in one unified sensor. First, the movement between an image and the next one was calculated based on feature point matching. The distance traveled on each image was calculated by summation. The process is shown in Figure 2 and was called the MISS1 method. Nevertheless, it was found that the accuracy of the MISS1 method was lower than that of the original MISS method in strain measurement.

**Figure 3:**
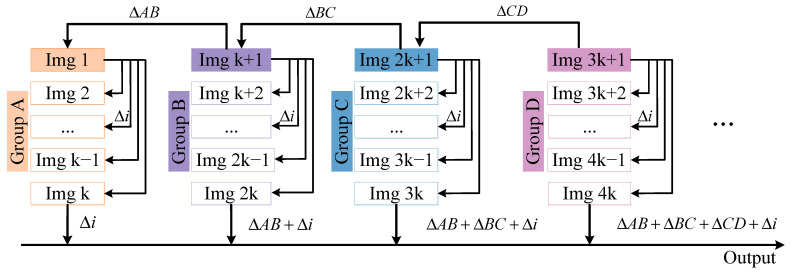
Process of the MISS100 method. Here, ∆i represents the movement from an image to the reference image in a feature group.

Then, the MISS100 method was developed to improve the accuracy and extend the measurement range. In the following, we define a feature group and describe how it is computed. Referring to Figure 3, a group is a set of *K* images; it included 100 images in this paper. Moreover, a larger integer can be set as long as the image can be captured within the initial view. Feature groups are essential to extend the measurement range and reduce the calculation time.

The first image of each group was selected as the reference image and was considered as the center of this group. In one group, each image was matched to the reference image. Then, the distance covered by the moving image could be calculated and expressed as ∆i. For different groups, like Group A and B, the first image of A was Img1, and B was Img (k + 1). The movement of the image in Group B was expressed as the sum of ∆i and ∆AB. ∆AB represents the difference in moving distance between two reference images in A and B. By that analogy, the moving distances of all images can be obtained by matching the movement of feature points. In general, the length of the image is not an integer multiple of *K*, and the remainder can be partitioned into the last feature group to be computed. In this way, the measured distance can be up to $L_{1}$, theoretically.

The pseudo algorithm of the matching algorithm (Algorithm 1) is as follows.
**Algorithm 1**: The high-level description of the MISS100 algorithm**PROCEDURE Match_MISS100**  **INPUT**: the length ***L*** of the sensor; the initial state ***preF = 1***, and ***delta = 0***; an intermediate state ***k***.  **OUTPUT**: ***strain*** obtained by the MISS100 method.   %%  **IF** the image length is divisible by 100, **THEN**     Match the image ***I*** and the image ***(I-99)*** based on the feature point matching algorithm     Obtain the distance ***move***     Update delta: ***delta = delta + move***      **For *k*** from ***(I-99)*** to ***I***        Match the image ***k*** and the image ***(I-99)*** based on the SURF algorithm        Obtain ***move2***        Calculate the movement of every image, ***sum***: ***sum = delta + move2***        Obtain strain according to ***sum*** and ***L***        Save ***strain***      **End**     Update ***perF = I-99***    **End** %% **IF** the image length is not divisible by 100, ***THEN***     Calculate the reminder ***N***, representing the number of images in the last group      Match the first image ***I1*** in the last group and the image ***(I-99)*** based on SURF     Obtain the distance ***move***     Update ***delta***: ***delta = delta + move***      **For** k from 1 to N        Match the image ***k*** and the image ***I1*** based on SURF        Obtain ***move3***        Calculate the movement of the remaining image, ***sum***: ***sum = delta + move3***        Obtain ***strain*** according to ***sum*** and ***L***        Save ***strain***      **End** **End**

## 4. Experiments Setup

**Figure 4:**
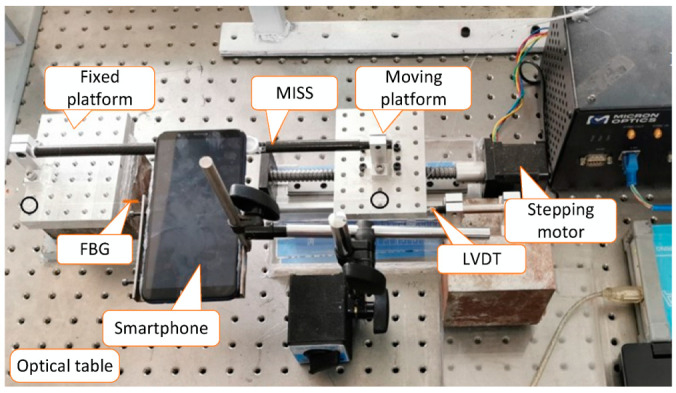
Schematic diagram of the field test arrangement.

To verify the effectiveness of the improved algorithm, both strain and displacement measurements were designed and performed. A schematic diagram of the field test arrangement is shown in Figure 4. The test equipment consisted of a smartphone, a mobile phone microscope, a MISS sensor, a stepper motor, a linear variable differential transformer (LVDT) and two platforms, one of which was fixed, and the other was moving. The length of the MISS sensor was 300 mm, and its left tube was about 105 mm long. More details of the MISS sensor were previously described [26,28]. The smartphone was a Honor 7X with 8 megapixels, and the microscope was a TIPSCOPE with thickness of 0.3 mm. The stepper motor had a length of 300 mm with a precision of 0.03 mm. in addition, the LVDT was DM8-02-5V, with a measurement range of 0–2 mm and an output voltage of 0 to 5 V.

### 4.1. Strain Measurement

For strain measurement, static and dynamic tests were performed previously as described [27,28]. In particular, in the static test, comparisons were based on images captured by smartphones with 8 megapixels, whereas in the dynamic test, they were carried out using videos recorded at 30 frames per second. All tests were carried out on a testing platform that was actuated by a stepper motor. In static tests, two work conditions were chosen, with step size of 5 μm and 10 μm. In the experiment, the moving platform was moved 10 times to the right and then 10 times to the left. That is, the maximum moving distances were 0.05 mm and 0.1 mm.

Similarly, the dynamic test was performed in two conditions with sinusoidal loading. The peaks of the sinusoidal waves were 0.05 mm and 0.1 mm, respectively. The waves were loaded by a program set up in the stepper motor. During the test, a smartphone was used to record videos, and then the images were extracted from the frames. An LVDT was adopted to acquire data for comparison.

### 4.2. Displacement Measurement

In addition, a displacement test was conducted to demonstrate the capabilities of the modified MISS method. The images were based on videos recorded by a smartphone with a resolution of 1920 × 1080 pixels. In the displacement tests, the step size of the movement was 50 μm or 100 μm. The maximum movements were 5 mm and 10 mm, after 100 or 200 moves. The stepper motor was directly used for comparison, since the distance was beyond the measurement range of the LVDT.

## 5. Result and Discussion

### 5.1. Static Tests for Strain Measurement

**Figure 5:**
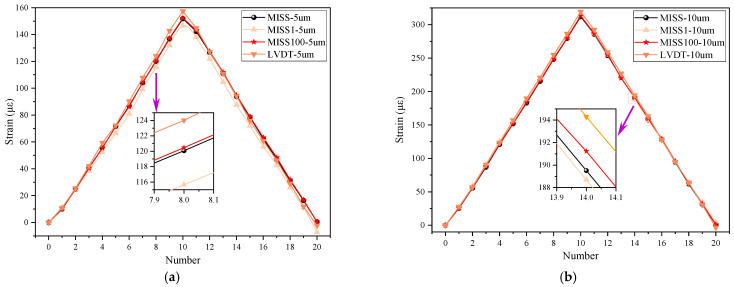
Comparison results of the static tests. (**a**) Comparative results for a movement of 5 μm; (**b**) comparative results for a movement of 10 μm.

To compare the accuracy of the MISS method and the improved methods, static tests were conducted, and the results are shown in Figure 5. Two working conditions were designed to test the performance of the improved method, with step size of 5 μm and 10 μm. A total of 420 images were used for the calculations in both tests. All results are plotted in Figure 5 for comparison. In the Figure, certain points were enlarged to display the details of the difference.

In Figure 5, all curves have the same trend, both on the ascent and on the descent, indicating that the improved method was effective. Moreover, the curve for the step size of 10 μm appeared better than that for the step size of 5 μm, because the former was more consistent. In the enlarged part in Figure 5a, the strain measured by the MISS100 method was similar to that obtained by the MISS method and close to the LVDT result. Furthermore, the strain calculated by the MISS1 method was the smallest. The same conclusion was reached when considering the enlarged view of Figure 5b. This showed that the difference was small when the step size was large. In addition, the data in the Figure suggest that the strain values in the two tests were 150 με and 300 με, respectively. This indicated that there exists a certain correlation between the two tests. The number of strains per unit of distance remained stable.

Besides, we analyzed the mean error, the standard deviation (SD), the root-mean-squared error (RMSE) and the maximum absolute errors of the differences obtained with the LVDT and the improved methods. The standard deviation is a measure of the amount of deviation of a set of values, and a low SD indicates that the values tend to be close to the mean of the set. The root-mean-squared error represents the difference between the values calculated by the improved method and those obtained with the LVDT.

As shown in Table 1, all errors calculated by the MISS1 method were larger when the step size was 5 μm. In addition, all errors obtained by the MISS1 method were the lowest, except for SD when using the step size of 10 μm. These results showed that the MISS1 method is greatly affected by the step size, and the erratic fluctuations were a consequence of the small step. On the whole, the results of the MISS and MISS100 methods were in good agreement, especially in the condition of a 5 μm step. The mean error, the SD, the RMSE and the maximum errors of the MISS100 method were 0.52, 2.96, 2.93, 5.53 με when the step was 5 μm. These results confirmed that the MISS100 method was effective without compromising the accuracy of the strain measurements.

### 5.2. Dynamic Tests for Strain Measurement

**Figure 6:**
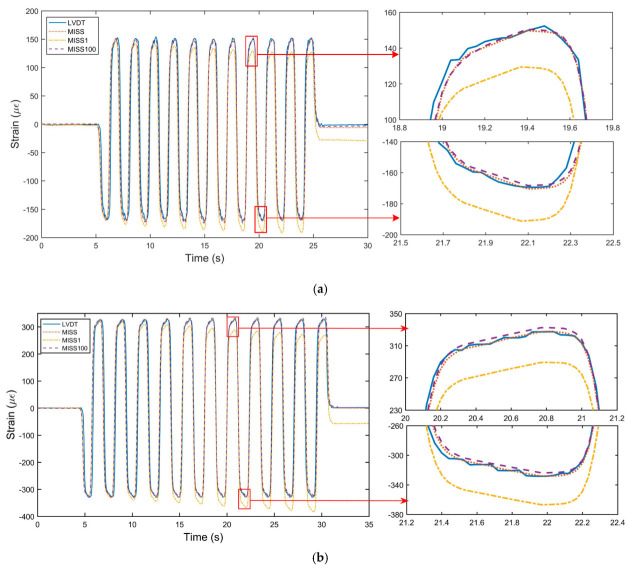
Comparison results of the dynamic tests. (**a**) Results for ±5 μm sin waves; (**b**) Results for ±10 μm sin waves.

To compare the stability of the MISS method and the improved method, dynamic tests were performed. Sin waves with an amplitude of 5 μm and 10 μm were adopted. We used 1677 and 1580 images for the calculations, respectively. In the same way, we reprocessed the images based on the MISS1 method and the MISS100 method. All results are plotted in Figure 6, where a part of the peaks is enlarged to display the details.

In Figure 6, all curves are in good agreement except for the MISS1 curve. Although the MISS1 curve had good consistency with respect to the LVDT measurements, the peak obtained with the MISS1 method deviated from that of the LVDT. The results calculated by the MISS1 method showed some cumulative errors when performing point-to-point image matching, which indicated that the errors were larger when using more groups. From the enlarged part in Figure 6a,b, it appeared that the strain measured by the MISS100 method was similar to that obtained by the MISS method. This demonstrated that the MISS100 method is effective and showed a limited error accumulation in the dynamic test.

Moreover, the error of the dynamic tests was analyzed, as shown in Table 2. As seen in the table, the parameters of the MISS1 method had much higher values than those of the other two methods. The SD and RMSE of the MISS100 method were close to and even lower than those of the MISS method. The results of these three methods indicated that the larger the group, the larger the errors, especially for the MISS1 method.

### 5.3. Tests for Displacement Measurement

**Figure 7:**
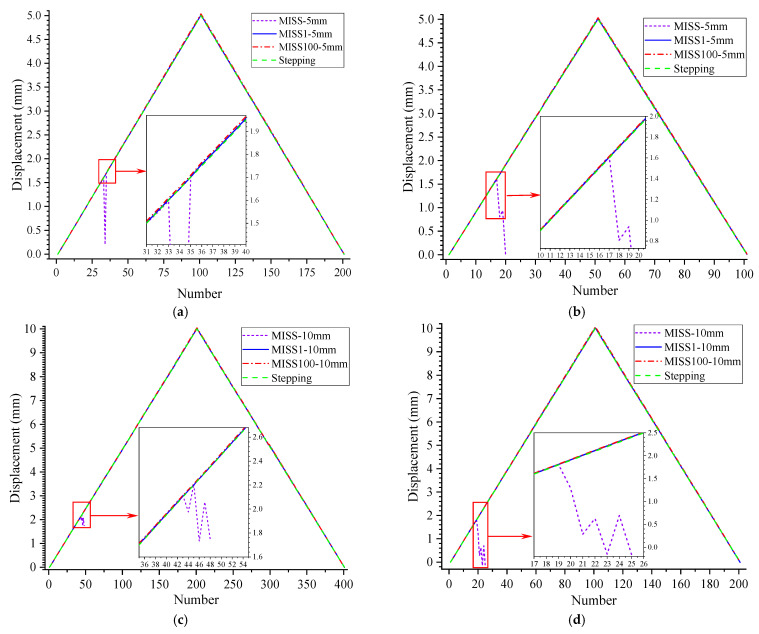
Comparison results of the displacement tests. (**a**) Displacement of 5 mm with a step of 50 μm; (**b**) displacement of 5 mm with a step of 100 μm; (**c**) displacement of 10 mm with a step of 50 μm; (**d**) displacement of 10 mm with a step of 100 μm.

To verify the capabilities of the improved MISS method, tests were performed to extend the measurement of displacement. We conducted four different sets of tests, with displacement and step size of 5 mm/50 μm, 5 mm/100 μm, 10 mm/50 μm and 10 mm/100 μm. Here, 50 μm and 100 μm was the distance traveled in each step, while 5 mm and 10 mm were the total distances. All results are plotted in Figure 7, and a part of the graphs is enlarged to display the details.

In Figure 7, the values calculated based on the MISS method appear to change greatly and then disappear, because the original feature points could not be recognized and were even out of view. Meanwhile, the curves obtained with the MISS1 and MISS100 methods were in good agreement, which indicated that the improved method could accurately measure the movement of the object. In Figure 7c, the peaks of the three curves obtained by MISS1, MISS100 and Stepping are 10.02, 10.06 and 10.00. The maximum difference was 0.06, which might be due to an inherent error in the stepping motor or in the algorithm. These small differences hardly affected the accuracy of the results obtained with the improved MISS method. As a whole, the measurement results did not show significant fluctuations or errors due to the variability of the step size and the measurement distance. This demonstrated that both the MISS100 and the MISS1 method were effective in the displacement test.

In strain measurement, the accumulation of the inherent error when using the MISS1 method needs to be tackled before its application. The larger the group, the larger the error. Within the range of observations, the images should be grouped as little as possible to avoid error accumulation. However, in displacement measurement, the MISS1 method was not affected by random errors, and its results were accurate. These results show that the strain and displacement measurements are differently prone to errors. In both dynamic and static tests for strain measurement, the MISS100 method based on SURF proved to be effective without losing accuracy.

## 6. Conclusions

In conclusion, a micro-image strain sensing (MISS) method was developed to measure strain in previous work and had a measurement range of ±8300 microstrains. However, the original MISS method has the disadvantage of a limited measurement range. In this paper, we propose a modified version of MISS, named MISS100, to solve the problem of the limited measurement range. Unlike the original MISS method, images were grouped, and strain was measured in an increased range with the MISS100 method. With the improvement, the theoretical measurement range can reach up to *L*_1_. Moreover, the method was also validated, showing good results for displacement and strain measurements. The results showed that the strain and displacement obtained by the MISS100 method correlated well with the LVDT data. This method can be readily applied to extend the measurement range for strain and displacement using one unified sensor. In the future, studies can focus on the optimization of the group size and the improvement of the MISS sensor. This research will likely stimulate the development of multifunctional sensors to obtain information on structures in the field of structural health monitoring.

## Figures and Tables

**Table 1:** Error analysis for the static tests.

Methods	Step Size (μm)	Mean Error (με)	SD (με)	RMSE (με)	Maximum Error (με)
MISS	5	0.9188	2.8020	2.8847	5.9343
MISS1	5	5.3023	3.2521	6.1795	10.8129
MISS100	5	0.5291	2.9607	2.9374	5.5308
MISS	10	3.4079	3.1223	4.5715	6.8128
MISS1	10	1.5590	3.5935	3.8378	5.9209
MISS100	10	2.6328	3.1142	4.0209	6.3073

**Table 2:** Errors analysis for the dynamic tests.

Methods	Step Size (μm)	Mean Error (με)	SD (με)	RMSE (με)	Maximum Error (με)
MISS	5	1.2258	1.1061	1.6342	2.7835
MISS1	5	15.6531	6.3752	16.8468	25.0418
MISS100	5	0.7706	1.2116	1.4125	2.6140
MISS	10	−0.8753	5.4329	5.3738	6.4823
MISS1	10	30.3059	18.5127	35.2824	59.9731
MISS100	10	−0.6642	5.2692	5.1850	−6.2224

## Data Availability

Not applicable.
